# Anxiety-associated factors among employees with different personality profiles: a cross-sectional study in China

**DOI:** 10.3389/fpsyg.2023.1043339

**Published:** 2023-06-22

**Authors:** Ran Hao, Jinfan Zuo, Haoyu Jin, Yipeng Wang, Lei Zhang, Yufang Zhu, Ye Wang, Yixin Qi, Jiale Qi, Jing Xia, Yibo Wu, Jie Hu

**Affiliations:** ^1^School of Nursing, Hebei Medical University, Shijiazhuang, Hebei, China; ^2^School of Public Health, Hebei Medical University, Shijiazhuang, Hebei, China; ^3^Department of Breast Center, The Fourth Hospital of Hebei Medical University, Shijiazhuang, Hebei, China; ^4^School of Journalism and Communication, Zhengzhou University, Zhengzhou, China; ^5^School of Nursing, North Sichuan Medical College, Nanchong, China; ^6^School of Public Health, Peking University, Beijing, China

**Keywords:** employees, anxiety, personality, latent profile analysis, self-efficacy, work-family conflict, perceived social support

## Abstract

**Introduction:**

Anxiety not only harms employees’ work efficiency and satisfaction but also presents as a hazard to their mental health. This study aimed to investigate the prevalence of anxiety among Chinese employees, identify their personality profiles and explore the anxiety-related factors in different personality profiles.

**Methods:**

This national investigation adopted the multistage random sampling method to recruit employees. A total of 3,875 employees were enrolled in this study, and 39.1% (1,515/3,875) of them were experiencing anxiety at the time of the study. Latent profile analysis (LPA) was conducted to identify personality subgroups among Chinese employees based on their BFI-10 scores.

**Results:**

LPA identified a three-profile solution among Chinese employees: average, resilient, and introverted. Employees in the resilient profile had the lowest anxiety rate (16.1%, 132/822), and those in the average profile had the highest rate (46.8%, 1,166/2,494). Multivariate analysis results showed that for all personality profiles, self-efficacy was positively associated with anxiety, and work-family conflict was negatively associated with anxiety. High levels of perceived social support and self-efficacy reduced the risk of anxiety and higher work-family conflict and no partner increased the odds of anxiety in the average profile. For the introverted profile, female gender, and living in a city increased the chances of suffering from anxiety.

**Discussion:**

This study identified that each personality profile of Chinese employees had its own set of factors associated with anxiety, which could facilitate employers to provide targeted interventions to alleviate employees’ anxiety.

## 1. Introduction

Anxiety, in the form of generalized anxiety disorder, is a common mental disorder among employees (Covid-19 Mental Disorders Collaborators, 2021). Studies showed that employees were reluctant to report mental disorders in the workplace for fear of experiencing stigma and unfair treatment (Barclay and Kiefer, 2019; Park and Kim, 2020). Disregarding anxiety could lead to difficulty in receiving a diagnosis and treatment. Furthermore, anxiety can reduce work efficacy, increase the probability of family conflict, and even lead to depression and suicide (Stanley et al., 2018; Kawohl and Nordt, 2020; Rasool et al., 2020). The coronavirus disease 2019 (COVID-19) outbreak has resulted in a continuous increase in the unemployment rate (International Labor Organization, 2020). During the COVID-19 pandemic, the risk of anxiety among employees would also rise with the growing unemployment rate (Lee et al., 2021). Hence, it is imperative to investigate the prevalence of anxiety among employees and the associated factors after the COVID-19 pandemic in China.

It is well known that particular personality traits are more commonly associated with the presence of anxiety disorders (Afshar et al., 2015). While numerous studies have examined the relationship between anxiety and the five basic dimensions of personality (Lee et al., 2020; Nikčević et al., 2021), these studies commonly examined the association between isolated personality traits and other factors using a variable-centered approach. This variable-centered approach neglects the mutual relationship between personality traits and humans interacting with environmental stimuli as a whole rather than a single personality trait (Li et al., 2021). Latent profile analysis (LPA) can address this problem effectively. This approach could provide insight into the mechanisms that produce both within-person variation and between-person differences across the observed dimensions (Isler et al., 2017). Several studies have used LPA to explore the subtypes of personality traits. Lan et al. (2021) identified two major personality profiles from hospitality employees (Lan et al., 2021). Semeijn et al. (2020) examined the latent profile of the 60-NEO PI-R in 293 employees in Netherlands and found two latent profiles named: the resilient profile and the introverted profile (Semeijn et al., 2020). Udayar et al. (2020) found three personality profiles among working adults in Switzerland named: the average profile, the resilient profile and the oversensitive profile (Udayar et al., 2020).

Factors that influence employee anxiety are multi-faceted. First, based on the conversation-of-resources (COR) theory, a conflict between work and family domains can drain emotional and physical resources leading to increased anxiety (Modaresnezhad et al., 2021). Work-family conflict (WFC) is a common problem that could be troubling for employees and maintaining the work-family balance can be difficult to do. Previous studies suggest that there is a relationship between WFC and anxiety (Obrenovic et al., 2020). Employees with high levels of WFC may be at a higher risk of anxiety (Zhang et al., 2020). Second, self-efficacy (SE) represents a person’s belief that he or she is capable of achieving a goal (Sherer et al., 1982), and reflects their ability to regulate external stress (Pierce et al., 1993). For employees, individuals with a high level of SE can quickly adapt to the changing workplace and high-force job demands, thus reducing their risk of anxiety (Chen et al., 2001). Third, according to the main effect model of social support (Cohen and Wills, 1985), the individual’s depression or anxiety can be directly reduced by the presence of social support. Perceived social support (PSS) refers to the way individuals perceive and assess actual or enacted social support (Sarason et al., 1990), which is known to be one of the most important factors in preventing mental disorders (Guo et al., 2021). High levels of PSS could, therefore, directly reduce anxiety in employees. Other influencing factors also include demographic characteristics (e.g., gender), economic status (e.g., monthly income), and interpersonal networks (e.g., marital and family status) (Karimi et al., 2020; Violant-Holz et al., 2020; Liu et al., 2021; Serapinas and Narbekovas, 2022). Given the highly personality-specific nature of anxiety and personal factors, disaggregation by personality is important. For example, nurses with normative and positive personality profiles were found to display higher self-efficacy and psychological resilience than those with negative personality profiles (Huang et al., 2021). Furthermore, individuals with highly adaptive profiles have been shown to have lower anxiety and depression compared to individuals with adaptive and maladaptive profiles (Li et al., 2021). To our knowledge, no previous study has categorized the latent profiles of Chinese employees based on the “Big Five Personality Traits” using a such large sample and explored factors influencing anxiety in different personality profiles.

Hence, this study aimed to identify Chinese employees’ personality profiles using LPA. Moreover, we analyzed the role of specific anxiety-associated factors in each personality profile among Chinese employees.

## 2. Materials and methods

### 2.1. Study design and participants

**FIGURE 1:**
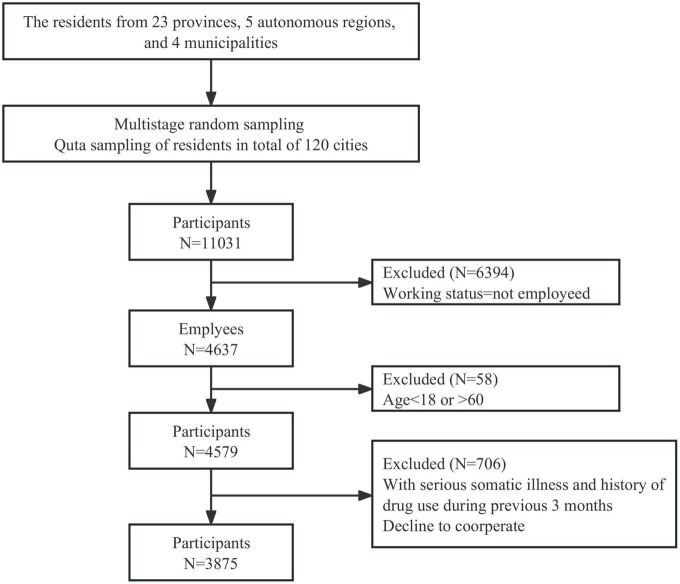
Flow diagram of participants enrollment.

In this study, we analyzed the anxiety of the participants using the Survey on Health Index Among Chinese Families (SHIACF2021) from July to September 2021. First, we randomly selected 120 cities in 23 provinces, five autonomous regions, and four municipalities directly under the central government. In the second stage, based on the results of the “Seventh National Census in 2021,” a quota sampling of 120 urban residents was conducted suggesting that the sample was representative of the population demographic in terms of gender, age, and urban-rural distribution. A total of 11,031 valid questionnaires were recovered. The questionnaires included Health China Action information, socio-demographic characteristics, and measurement scales. The inclusion criteria for participants were (i) currently working, (ii) aged between 18 and 60 years old, and (iii) complete the consent form. The exclusion criteria included having serious somatic or psychiatric disorders. Consequently, a total of 3,875 employees were included in this study (Figure 1).

### 2.2. Ethical considerations

Study participants signed informed consent and this study was approved by the Ethics Committee of Jinan University, Guangzhou, China (JNUKY-2021-018).

### 2.3. Measurements

#### 2.3.1. General characteristics

General characteristics included age, gender, occupation, marital status, monthly income, number of siblings, number of properties, and debts.

#### 2.3.2. Anxiety

The generalized anxiety disorder (GAD-7) (Spitzer et al., 2006) was applied to detect anxiety, using seven items scored on a four-point Likert scale (0–3) (Mossman et al., 2017). The total score was obtained by adding all the items together, with a higher score indicating a more severe anxiety symptom. In this study, a score of less than five indicated no anxiety (Hinz et al., 2017). Cronbach’s alpha for this study was 0.951.

#### 2.3.3. Self-efficacy (SE)

The new general self-efficacy scale (NGSES) (Chen et al., 2001) contains eight items that are scored on a 5-point scale (1 = strongly disagree, 5 = strongly agree). The total score was obtained as an average of the eight items, with a higher score indicating greater SE (Salles, 2017). Cronbach’s alpha was 0.941 in this study.

#### 2.3.4. Work-family conflict (WFC)

The work-family conflict scale (WFCS) (Haslam et al., 2015) contains two dimensions: work-to-family conflict and family-to-work conflict. Five items for each dimension were rated on a 7-point scale (1 = very strongly disagree, 7 = very strongly agree). Each dimension was scored according to the sum of its items, and the total score was determined based on the sum of each dimension’s points, with higher scores representing higher levels of WFC. Cronbach’s alpha for this study was 0.944.

#### 2.3.5. Perceived social support (PSS)

The perceived social support scale (PSSS) consists three dimensions, including family support, friends support, and other support (Zimet et al., 1990). Items were rated on a seven-point scale from 1 = very strongly disagree to 7 = very strongly agree. The total score was calculated as the sum score of all dimensions scores. In this study, Cronbach’s alpha was 0.960.

#### 2.3.6. Personality

The 10-item short version of the Big Five Inventory (BFI-10) includes extraversion, agreeableness, conscientiousness, neuroticism, and openness (Rammstedt and John, 2007). Items were rated on a five-point scale from 1 = strongly disagree to 5 = strongly agree. The scores of extraversion were summed of the scores of item 1R and item 6, the scores of agreeableness were combined with the scores of items 2 and 7R, the scores of conscientiousness as 3R and 8, neuroticism as 4R and 9, and openness as 5R and 10 (*R* = item is reversed-scored). The Cronbach’s alpha for extraversion, agreeableness, conscientiousness, neuroticism, and openness were 0.723, 0.759, 0.786, 0.753, and 0.714, respectively (Wang et al., 2014).

## 3. Statistical analysis

IBM SPSS 26.0 and M-plus 8.0 were used for analyses. First, we performed latent profile analysis (LPA) to identify latent profiles of employees based on their BFI-10 scores using M-plus 8.0. The scores of sub-scales (openness, agreeableness, neuroticism, conscientiousness, and extraversion) in BFI-10 were used as explicit indicators. A model-fitting process begins with a one-profile model to which additional profiles were added one at a time. To choose between competing models, we used a variety of indicators: akaike information criteria (AIC), Bayesian information criteria (BIC), adjusted Bayesian information criteria (aBIC), entropy, and Lo-Mendell-Rubin adjusted likelihood ratio test (LMRT). The lowest values of AIC, BIC, and aBIC indicated the model with the best fit. In general, entropy value ranges from 0 to 1, with values approaching 1 indicating a clear description of the classes. The LMRT test (*P* < 0.05) indicated that a model with a k profile provided a better fit than one with a k-1 profile.

Second, the next analyses were performed separately for each personality profile. The median [interquartile range (IQR)] was used for continuous variables. The categorical variables were expressed as frequency numbers (percentages). As appropriate, univariate analysis was performed using the Wilcoxon rank-sum or chi-square test. Multivariate analysis was performed using binary logistic regression analysis (Forward, likelihood ratio method). *P*-value < 0.05 was considered statistically significant.

## 4. Results

### 4.1. Characteristics of employees

The demographic information of employees is presented in Supplementary Table 1. In total, 3,875 employees were included in this study (47.7%, 1,849 male participants). The employees included in this study were separated into seven categories according to the People’s Republic of China Occupational Classification Dictionary. A total of 1,027 (26.5%) participants were professional and technical staff, and 922 (23.8%) participants were other practitioners. Most (83.4%, 3,230) of the employees resided in a city (Supplementary Table 1). The median scores of other scales were listed as follows: the NGSES score was 3.875 (IQR = 3.125–4.000), the PSSS score was 61.00 (IQR = 51.00–72.00), and the WFCS score was 25.00 (IQR = 20.00–30.00). The median GAD-7 of all employees was 3.00 (IQR = 0.00–7.00), with 39.1% (1,515/3,875) receiving scores indicative of anxiety (GAD-7 ≥ 5).

### 4.2. Personality trait profiles of employees

Five types of latent profile models of the participants’ personalities were constructed by LPA. A summary of the LPA fit indices for the one- to five-profile models is presented in Table 1. The four- and five-profile solutions had lower AIC, BIC, and aBIC values than the two- and three-profile solutions. Of these, the three-profile model represented the best performance and had a significant LMR value (*P* = 0.0014). In contrast, LMR values of the four-profile and five-profile were not significant, providing further support to the three-profile. Meanwhile, the profile sizes of the three-profile solution were satisfactory, ranging between 14.4 and 64.4%. Based on the fit indices, the three-profile solution was considered the most adequate.

**TABLE 1:** Model fit indices for the latent profile analysis (*N* = 3,875).

Profile	K	Log-likelihood	AIC	BIC	aBIC	Entropy	*p*LMR	*p*BLRT
1	10	−35,942.357	71,904.713	71,967.336	71,935.561			
2	16	−35,237.439	70,506.877	70,607.074	70,556.234	0.720	< 0.0001	< 0.0001
**3**	**22**	−**35,070.385**	**70,184.771**	**70,322.541**	**70,252.636**	**0.681**	**0.0014**	**0.0016**
4	28	−34,947.696	69,951.392	70,126.737	70,037.766	0.752	0.2104	0.2160
5	34	−34,831.417	69,730.835	69,943.753	69,835.717	0.763	0.0633	0.0663

Values in bold indicate the best fitting model. K, number of parameters; AIC, akaike information criterion; BIC, Bayesian information criterion; *p*LMR, *p*-values for Lo-Mendell-Rubin adjusted likelihood ratio test for K vs. K-1 profiles; *p*BLRT, *p*-values for bootstrapped likelihood-ratio test.

**FIGURE 2:**
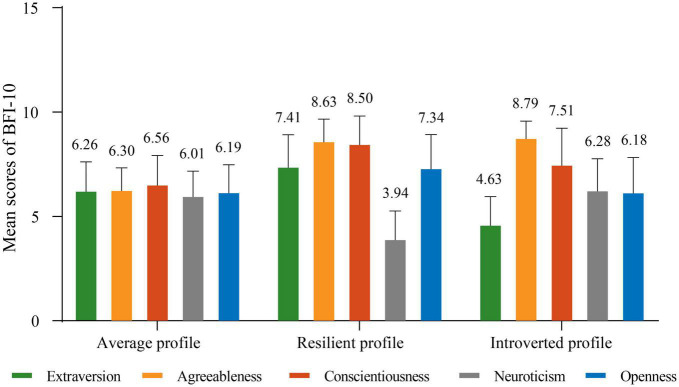
Distributions of mean scores of BFI-10 in the three profiles of Chinese employees.

The three-profile solution is presented in Figure 2. The largest profile (64.4%, 2,494/3,875) scored almost equally on all personality traits. This group was defined as the “average profile.” The second-largest profile (21.2%, 822/3,875) presented higher extraversion and openness but lower neuroticism. This group was labeled the “resilient profile.” The last profile (14.4%, 559/3,875) showed lower extraversion and openness, but higher neuroticism. This group was named the “introverted profile.” The detail of significant differences between latent profile classes for the personality dimensions is presented in Supplementary Table 2.

### 4.3. Univariate analysis of anxiety in different personality profiles

We compared the incidence of anxiety (GAD-7 ≥ 5) among the three profiles (Table 2). The results indicated that the participants categorized in the resilient profile had the lowest anxiety rate (16.1%, 132/822) and those categorized in the average profile had the highest rate (46.8%, 1,166/2,494). Next, the incidence of anxiety in the three extracted employees’ profiles was compared in terms of relevant scales (Supplementary Table 3). The findings indicated that, in the average and introverted profile, employees with anxiety had lower levels of SE (*p* < 0.001) and PSS (*p* < 0.001) and higher levels of WFC (*p* < 0.001) compared with employees who did not experience anxiety. Yet in the resilient profile, the difference in PSS levels between employees with anxiety and those who did not experience anxiety was not significant (*p* = 0.079) (Supplementary Table 3).

**TABLE 2:** *Post-hoc* test among personality traits profiles in anxiety (*N* = 3,875).

Group	Average profile (*N* = 2,494)	Resilient profile (*N* = 822)	Introverted profile (*N* = 559)	χ ^2^	*p*-value
Anxiety^#^	1,166 (46.8%)	132 (16.1%)	217 (38.8%)	244.631	< 0.001
No-anxiety	1,328 (53.2%)	690 (83.9%)	342 (61.2%)		

^#^GAD-7 scores ≥ 5. Differences among three personality profiles were all significant.

### 4.4. Multivariable analysis of anxiety in different personality profiles

To explore the independent factors associated with anxiety in each personality trait profile, a multivariate binary logistic regression analysis was performed. The results of logistic regression analysis in different personality trait profiles are shown in Tables 3–5, respectively. In the fully adjusted model, for the average profile, high SE (OR 0.749, 95% CI 0.633–0.888) and PSS (OR 0.989, 95% CI 0.980–0.997) decreased the risk of anxiety (Table 3). Conversely, the higher WFC (OR 1.122, 95% CI 1.108–1.136) and no partner (Unmarried OR 1.305, 95% CI 1.054–1.615; divorced/widowed OR 1.746 95% CI 1.032–2.956) increased the odds of anxiety (Table 3). In the resilient profile, the results showed that SE (OR 0.611, 95% CI 0.427–0.875) was a protective factor for anxiety, while WFC (OR 1.109, 95% CI 1.082–1.138) was a risk factor for anxiety (Table 4). For the introverted profile, a high SE (OR 0.569, 95% CI 0.400–0.808) was associated with a reduced risk of anxiety (Table 5). Additionally, high WFC (OR 1.111, 95% CI 1.081–1.143), female gender (OR 2.284, 95% CI 1.540–3.385), and living in a city (OR 1.862, 95% CI 1.062–3.264) were independent risk factors for anxiety (Table 5).

**TABLE 3:** Forward likelihood ratio stepwise logistic regression of risk factors for anxiety in the average profile (*N* = 2,494).

Factors	Wald	OR (95% CI)	*p*-value
SE	11.15	0.749 (0.633–0.888)	0.001
WFC	315.97	1.122 (1.108–1.136)	< 0.001
PSS	6.50	0.989 (0.980–0.997)	0.011
Marital status			0.009
Married		1 (Ref)	
Unmarried	5.97	1.305 (1.054–1.615)	0.015
Divorced/widowed	4.31	1.746 (1.032–2.956)	0.038

SE, self-efficacy; WFC, work-family conflict; PSS, perceived social support; OR, odds ratio; 95% CI, 95% confidence interval.

**TABLE 4:** Forward likelihood ratio stepwise logistic regression of risk factors for anxiety in the resilient profile (*N* = 822).

Factors	Wald	OR (95% CI)	*p*-value
SE	7.23	0.611 (0.427–0.875)	0.007
WFC	64.82	1.109 (1.082–1.138)	< 0.001

SE, self-efficacy; WFC, work-family conflict; OR, odds ratio; 95% CI, 95% confidence interval.

**TABLE 5:** Forward likelihood ratio stepwise logistic regression of risk factors for anxiety in the introverted profile (*N* = 559).

Factors	Wald	OR (95% CI)	*p*-value
SE	9.916	0.569 (0.400–0.808)	0.002
WFC	55.181	1.111 (1.081–1.143)	< 0.001
**Gender**			
Male		1 (Ref)	
Female	16.90	2.284 (1.540–3.385)	< 0.001
**Residence**			
Rural		1 (Ref)	
City	4.71	1.862 (1.062–3.264)	0.030

SE, self-efficacy; WFC, work-family conflict; OR, odds ratio; 95% CI, 95% confidence interval.

## 5. Discussion

### 5.1. Theoretical and research implications

Findings from the national survey in China indicated that the prevalence of anxiety among employees was 39.1%, which was higher than that of employees in European or Australian countries (13–35%) (Gémes et al., 2022). Based on the reciprocal determinist model, an individual’s behavior is influenced by both personal and social factors (Bandura, 1990), therefore factors influencing anxiety are also different. Importantly, researchers have identified that personality serves a key role in anxiety. However, previous studies have considered personality traits individually (Ka et al., 2021; Nikčević et al., 2021) and ignored the fact that people are composed of various personality patterns. To address this knowledge gap, this study performed LPA to explore the personality profiles of Chinese employees and analyzed the factors influencing anxiety in different personality profiles.

According to the LPA results, we divided Chinese employees into three profiles according to their Big Five personality traits, namely the average profile, the resilient profile, and the introverted profile (Figure 2 and Supplementary Table 2). The average profile was characterized by relatively low scores on agreeableness, conscientiousness, and openness but moderate scores on extraversion and neuroticism. In contrast, the introverted profile showed the highest scores on neuroticism and agreeableness but the lowest scores on extraversion. The resilient profile represented low scores on neuroticism, but average to high scores on the four other traits. We found that employees in the average profile had the highest prevalence of anxiety (46.8%, 1,166/2,494), followed by the introverted profile (38.8%, 217/559), and the lowest prevalence was in the resilient profile (16.1%, 132/822). Similarly to previous studies, individuals in the under-controlled profile (i.e., average profile) were at risk for mental illness (Donnellan and Robins, 2010). Min and Su (2020) depicted that the resilient profile has the lowest rate of work-related burnout compared to other profiles (Min and Su, 2020). The resilient profile contained the ideal personality traits, which made it the profile least likely to be associated with anxiety. According to a meta-analysis, positive affect was predicted equally well by extraversion and agreeableness (Bucher et al., 2019). Meanwhile, neuroticism was the strongest predictor of negative effects (Bucher et al., 2019). In our study, individuals within the average profile scored low on both agreeableness and extraversion but moderate on neuroticism, which caused the average profile to have the highest rates of anxiety. Individuals within the introverted profile scored high on neuroticism and agreeableness, but low on extraversion, leading to this profile having the second highest anxiety level only after the average profile. This phenomenon reflects the fact that various personality types are subject to anxiety to different degrees identifying the innate effect of personality on anxiety.

To explore the causes of the employees’ anxiety in each profile, we conducted a multivariable regression analysis. Results showed that both SE and WFC were the independent predictive factors for the employees’ anxiety in all profiles. SE is an important resource for coping with anxiety (Bakker et al., 2010). Employees with a higher level of SE exhibit a greater level of confidence to execute new tasks (Bandura, 1997). Therefore, employees with higher SE were less likely to experience anxiety. Furthermore, SE can reduce work-related stress and anxiety (Lange and Kayser, 2022) and is negatively correlated with anxiety (Simonetti et al., 2021).

Moreover, our results indicated that regardless of the personality profile of the individual, WFC was a significant factor in anxiety, which confirmed the COR theory. WFC is a form of inter-role conflict (Wharton, 1993) that occurs when work demands interfere with a person’s private life (Barbieri et al., 2021) and has been linked to symptoms of anxiety (Zhang et al., 2020; Modaresnezhad et al., 2021), particularly in women (Frank et al., 2021). Additionally, WFC is positively related to anxiety among employees specifically (Frone, 2000). COR theory states that individuals have limited mental, emotional, and physical resources (Hobfoll, 1989). Taking on too many roles at work or in a family can lead to anxiety because of the inability to fulfill the expectations of the other role. Our results were consistent with this theory.

Perceived social support was a protective factor against anxiety only in the average profile employees. Prior studies have demonstrated that PSS is a protective factor for anxiety (Chen et al., 2021; Guo et al., 2021), and it is more beneficial to an individual’s mental health than objective support (Park et al., 2014; Wahab et al., 2021). This was consistent with our results in participants with the average profile and the main effect model of social support. However, we did not find the association between PSS and anxiety in participants with resilient and introverted profiles (Tables 4, 5). The above result contradicts the main effect model of social support. We deduced that there were two reasons. The first possible reason was that the employees in the resilient (8.63 ± 1.03) and introverted profiles (8.79 ± 0.78), possessed higher levels of agreeableness compared with those in the average profile (6.30 ± 1.03). Agreeableness could increase the endogenous appreciation of exogenous social support (Barańczuk, 2019). This leads to an overall higher perception of external social support in both profiles, with less variation between anxiety and non-anxiety. Cohen and Wills (1985) speculate that reducing mental health problems is dependent on a threshold of social support (Cohen and Wills, 1985). Once this threshold is reached, any amount of social support will not help. Therefore, the main effect of perceived social support on the alleviation of anxiety disappeared among the employees with resilient and introverted profiles (those who were at high levels of PSS overall in the results of this study).

The second possible reason was that as found by Lakey and Cassady (1990), the association between perceived social support and psychological distress could be explained by individual differences in negative cognition (Lakey and Cassady, 1990). To understand the relationship between perceived social support and anxiety, personality factors need to be considered. Meanwhile, Luszczynska and Cieslak (2005) also proved that only those with high emotion reactivity would benefit more from social support (Luszczynska and Cieslak, 2005). Our results were consistent with these findings. Furthermore, employees with resilience and introverted profiles scored higher on the conscientiousness dimension, which was positively correlated with self-control (Zhou et al., 2021), so they were less emotionally reactive. They were less likely to benefit from PSS, thus, PSS did not serve as a protective factor for anxiety in these two profiles (resilience and introverted). Our results indicated that the main effect model of social support was suitable for individuals with the average profile.

For participants with the introverted profile, the female gender was an independent risk factor for higher anxiety among employees. This was consistent with prior studies that focused on the relationship between gender and anxiety (Nguyen et al., 2019; Gao et al., 2020). According to the results of Catuzzi and Beck (2014) anxiety may be exacerbated by the two-hit model in women (Catuzzi and Beck, 2014). They found that women seem more likely to suffer from anxiety disorders if the stimulus-response associations were more rigid (Catuzzi and Beck, 2014). Employees with the introverted profile had lower scores for extroversion and openness, which were connected to cognitive flexibility (Smith and Konik, 2022). This might lead to women only with the introverted profile suffering from anxiety. Furthermore, living in a city was also an independent risk factor for anxiety in employees with an introverted profile. That may be because employees working in the city experienced higher levels of job stress and economic pressure. Thus, more attention should be paid by employers to mitigate the risk of anxiety.

This study has several limitations. First, our study is a cross-sectional design, which makes it impossible to draw conclusions about causal relationships between anxiety and its associated factors. Second, the entropy of LPA in this study is 0.681. According to the study of Clarke and Kissane (2002) entropy between 0.6 and 0.8 is moderate classification accuracy. So, the three-profile solution adopted in this study was acceptable.

### 5.2. Practical implications

Our study has certain practical implications. First, human-resource development should take the ability of SE, PSS, and the ability to balance work and family into consideration when assessing employees. Second, to reduce the employees’ anxiety, supervisors could support employees to improve their SE level by encouraging them to participate in decision-making processes and to relieve WFC by supplying periodic psychosocial counseling. Leader support and psychological support in the workplace have been shown to reduce employees’ WFC (French et al., 2018). Third, supervisors must attend training to learn how to detect anxiety-related factors, as well as how to offer support to employees.

To our knowledge, this is the first study to investigate Chinese employees’ personality profiles, identify the three-profile solution, and investigate the factors influencing the anxiety of employees with different personality profiles. Second, in contrast to previous variable-centered research methods, the personality-LPA approach considers the interactive combination of each personality trait and views the individual as a whole in which all dimensions of personality coexist, maximizing the heterogeneity among employees. Moreover, the findings of this study could assist supervisors in developing targeted interventions based on the anxiety influencing factors in different personality profiles, which will not only benefit employees’ mental health but also contribute to the organization’s development.

## 6. Conclusion

High rates of anxiety among Chinese employees were identified in this study. Based on the Big Five personality traits, Chinese employees were categorized into three profiles: the average profile, the resilient profile, and the introverted profile. Furthermore, our results demonstrated that SE and WFC remain influential factors of anxiety among employees with different personality profiles, while the main effect of social support on anxiety needs to be in employees with the average profile. Finally, our study will assist management in identifying at-risk populations and implementing appropriate interventions.

## Data availability statement

The original contributions presented in this study are included in the article/Supplementary material, further inquiries can be directed to YBW, bjmuwuyibo@outlook.com.

## Author contributions

JH, YBW, and RH designed the research. RH, JZ, HJ, YPW, LZ, YZ, YeW, JQ, and JX collected and analyzed the data. RH, JZ, and HJ wrote the manuscript. YQ revised the manuscript. All the authors contributed to the article, read, and approved the final manuscript.
