# Supplementary material for: Anxiety-associated factors among employees with different personality profiles: a cross-sectional study in China
Source: Front Psychol. 2023 Jun 22;14:1043339. doi: 10.3389/fpsyg.2023.1043339 (PMC10325660; doi:10.3389/fpsyg.2023.1043339)
Supplement: Supplementary file 1 [file Data_Sheet_1.docx]

Supplementary Material

# Supplementary Tables

| TABLE S1 Characteristics of the participants (N = 3875). | |  |
| --- | --- | --- |
| Variables | N (%) or Median (IQR) | Code |
| Gender |  |  |
| Male | 1849 (47.7%) | 1(Ref) |
| Female | 2026 (52.3%) | 2 |
| Age |  |  |
| 18-30 | 1089 (28.1%) | 1(Ref) |
| 31-40 | 1141 (29.4%) | 2 |
| 41-50 | 1296 (33.4%) | 3 |
| 50-60 | 349 (9.0%) | 4 |
| Residence |  |  |
| Rural | 645 (16.6%) | 1(Ref) |
| City | 3230 (83.4%) | 2 |
| Number of siblings |  |  |
| Zero | 877 (22.6%) | 1(Ref) |
| One | 1188 (30.7%) | 2 |
| ≥Two | 1810 (46.7%) | 3 |
| Marital status |  |  |
| Married | 3031 (78.2%) | 1(Ref) |
| Unmarried | 741 (19.1%) | 2 |
| Divorced/Widowed | 103 (2.7%) | 3 |
| Number of properties |  |  |
| Zero | 293 (7.6%) | 1(Ref) |
| One set | 2335 (60.3%) | 2 |
| ≥Two sets | 1247 (32.2%) | 3 |
| Debt |  |  |
| No | 2247 (58.0%) | 1(Ref) |
| Yes | 1628 (42.0%) | 2 |
| Monthly income  (yuan) |  |  |
| ≤3000 | 662 (17.1%) | 1(Ref) |
| 3001-6000 | 1624 (41.9%) | 2 |
| 6001-9000 | 1589 (41.0%) | 3 |
| Occupation |  |  |
| Type 1 | 340 (8.8%) | 1(Ref) |
| Type 2 | 746 (19.3%) | 2 |
| Type 3 | 1027 (26.5%) | 3 |
| Type 4 | 201 (5.2%) | 4 |
| Type 5 | 517 (13.3%) | 5 |
| Type 6 | 122 (3.1%) | 6 |
| Type 7 | 922 (23.8%) | 7 |
| SE | 3.875 (3.125-4.000) |  |
| PSS | 61.00 (51.00-72.00) |  |
| WAFC | 25.00 (20.00-30.00) |  |
| GAD-7 | 3.00 (0.00-7.00) |  |
| IQR, Interquartile range; SE, Self-efficacy; PSS, Perceived Social Support; WAFC, Work-Family Conflict; GAD-7, The 7-Item Generalized Anxiety Disorder; Type 1, Clerical and related personnel; Type 2, Personnel of state institutions; Type 3, Professional technician; Type 4, Production, operation, and service personnel; Type 5, Business and services personnel; Type 6, Agribusiness, forestry, animal husbandry, and fishery personnel; Type 7, Other workers. | | |

| Table S2 Mean differences in personality traits between latent profiles. | | | | | | | | | |
| --- | --- | --- | --- | --- | --- | --- | --- | --- | --- |
| Personality traits | Latent profile classes | | | | | | F | post hoc | |
|  | Average profile  (N = 2494) | | Resilient profile  (N = 822) | | Introverted profile  (N = 559) | |  |  |  |
|  | M | SD | M | SD | M | SD |  |  |  |
| Extraversion | 6.26 | 1.35 | 7.41 | 1.5 | 4.63 | 1.32 | 675.88 | 2>1>3 |  |
| Agreeableness | 6.30 | 1.03 | 8.63 | 1.03 | 8.79 | 0.78 | 2565.71 | 3>2>1 |  |
| Conscientiousness | 6.56 | 1.36 | 8.50 | 1.31 | 7.51 | 1.72 | 616.12 | 2>3>1 |  |
| Neuroticism | 6.01 | 1.16 | 3.94 | 1.32 | 6.28 | 1.48 | 952.33 | 3>1>2 |  |
| Openness | 6.19 | 1.28 | 7.34 | 1.58 | 6.18 | 1.64 | 219.57 | 2>1 2>3 |  |
| 1, Average profile; 2, Resilient profile; 3, Introverted profile. **p* < 0.05, ***p* < 0.01, ****p* < 0.001. | | | | | | | | | |

| TABLE S3 Univariable analysis for factors associated with anxiety in each profile among Chinese employees. | | | | | | | | | |
| --- | --- | --- | --- | --- | --- | --- | --- | --- | --- |
|  | Average profile  (N = 2494) | |  | Resilient profile  (N = 822) | |  | Introverted profile  (N = 559) | |  |
|  | Non-anxiety  median (IQR) | Anxiety  median (IQR) | *Z* | Non-anxiety  median (IQR) | Anxiety  median (IQR) | *Z* | Non-anxiety  median (IQR) | Anxiety  median (IQR) | *Z* |
| PSS | 60.00  (49.00-69.00) | 55.00  (48.00-64.00) | －6.81^***^ | 71.00  (63.00-77.00) | 70.50  (61.00-73.00) | －1.75 | 67.00  (59.00-72.00) | 62.00  (53.00-70.50) | －4.2^***^ |
| SE | 3.75  (3.00-4.00) | 3.50 (3.00-4.00) | －6.3^***^ | 4.00  (3.88-4.50) | 4.00  (3.75-4.00) | －3.63^***^ | 3.88  (3.50-4.00) | 3.75  (3.12-4.00) | －4.26^***^ |
| WAFC | 23.00  (19.00-30.00) | 30.00  (26.00-33.00) | －19.83^***^ | 20.00  (14.00-24.00) | 26.00  (20.00-31.00) | －8.81^***^ | 21.00  (17.00-27.00) | 28.00  (22.50-30.50) | －8.07^***^ |
| IQR, Interquartile range; PSS, Perceived Social Support; SE, Self-efficacy; WAFC, Work-Family Conflict. **p* < 0.05, ***p* < 0.01, ****p* < 0.001. | | | | | | | | | |
